# Gestational Alcohol Exposure Altered DNA Methylation Status in the Developing Fetus

**DOI:** 10.3390/ijms18071386

**Published:** 2017-06-28

**Authors:** Chanchal Mandal, Debasish Halder, Kyoung Hwa Jung, Young Gyu Chai

**Affiliations:** 1 Department of Molecular and Life Science, Hanyang University, 15588 Ansan, Korea; chanchalbge@gmail.com (C.M.); deba_genetic@yahoo.com (D.H.); khjung2@gmail.com (K.H.J.); 2 Institute of Natural Science and Technology, Hanyang University, 15588 Ansan, Korea; 3 Department of Bionanotechnology, Hanyang University, 04763 Seoul, Korea

**Keywords:** alcohol, epigenetics, DNA methylation, FASD, fetal development

## Abstract

Ethanol is well known as a teratogenic factor that is capable of inducing a wide range of developmental abnormalities if the developing fetus is exposed to it. Duration and dose are the critical parameters of exposure that affect teratogenic variation to the developing fetus. It is suggested that ethanol interferes with epigenetic processes especially DNA methylation. We aimed to organize all of the available information on the alteration of DNA methylation by ethanol in utero. Thus, we have summarized all published information regarding alcohol-mediated alterations in DNA methylation during gestation. We tried to arrange information in a way that anyone can easily find the alcohol exposure time, doses, sampling time, and major changes in genomic level. Manuscript texts will also represent the correlation between ethanol metabolites and subsequent changes in methylome patterns. We hope that this review will help future researchers to further examine the issues associated with ethanol exposure.

## 1. Introduction

It has long been believed that the ingestion of alcohol during pregnancy can lead to the birth of infants with developmental defects. The forms of abnormalities resulting from ethanol exposure vary depending on the dose, duration, and frequency of consumption during the gestational period. Maternal genetics and metabolism are also important factors. Alcohol is well known as a teratogenic agent that affects the normal development of a developing embryo. Fetal alcohol spectrum disorder (FASD) is the term commonly used to denote alcohol-related neonatal abnormalities [1,2]. The most severe form of FASD is known as fetal alcohol syndrome (FAS), and manifests as growth retardation, facial abnormalities, and central nervous system (CNS) deficiencies [3,4]. There is no specific time point during gestation when alcohol exposure is not accompanied by harmful consequences. However, the most severe birth defects are correlated with alcohol exposure during the embryonic stage rather than later fetal stages [5]. FAS and other alcohol-related birth defects are examples of what can happen when a mother ingests alcohol during her pregnancy [6]. Many previous studies have described genetic susceptibilities to alcohol-mediated defects. Microarray analysis and next-generation sequencing techniques have found that many developmental genes are altered by in utero alcohol exposure [1,2,3,4,7,8,9,10]. However, the underlying causes of these alcohol-mediated disorders remain unclear. A combination of genomic, genetic, and epigenetic alcohol research could reveal the molecular mechanisms of these disorders, adding to the utility of investigating the epigenetic and genetic factors involved in ethanol-mediated birth defects.

**Figure 1:**
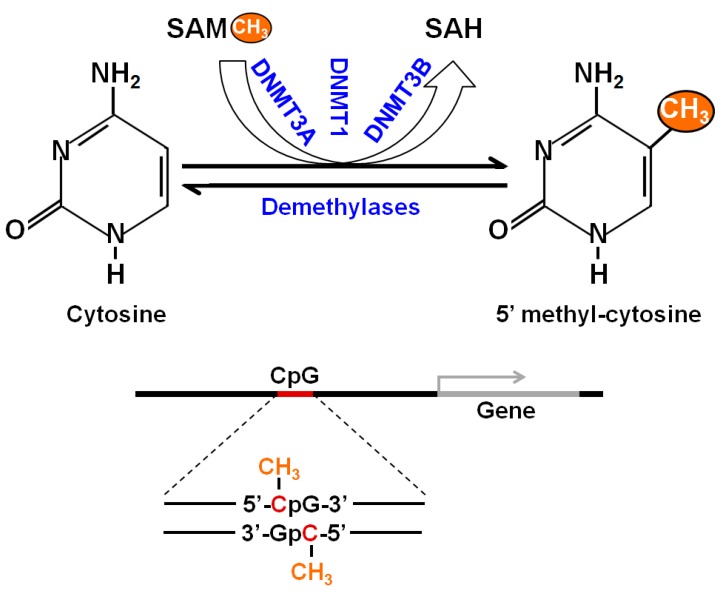
Schematic representation of DNA methylation. The process starts with the covalent addition of a methyl group to form 5-methylcytosine (5-mC). This process is catalyzed by a family of DNA methyltransferases (DNMTs)—DNMT1, DNMT3A and DNMT3B. The majority of DNA methylation usually occurs at CpG sites and CpG islands nearby a gene to regulate related gene expression. DNA methylation is an epigenetic mechanism and required fine tuning for proper regulation.

Epigenetics is the study of changes in gene expression that do not involve changes to the underlying DNA sequence. These changes affect cells’ phenotypic expression by regulating relative gene expression levels [11]. Epigenetic effects are a common and natural process in living cells, and they occur under tightly controlled and pre-programmed mechanisms. Epigenetics can also be influenced by factors including environmental conditions, age, lifestyle, exposure to toxicants, and disease state. Epigenetic changes are usually non-inherited, but it may be possible to pass down epigenetic changes to offspring if the changes occur in sperm or egg cells. Most epigenetic changes occurring in sperm and egg cells are deleted when the cells combine to form a zygote. Epigenetic regulations are mediated through DNA methylation, histone modification, and non-coding RNA (ncRNA)-associated gene silencing [12,13,14]. DNA methylation occurs via the covalent addition of a methyl group (CH_3_) at the 5-carbon of the cytosine ring, resulting in 5-methylcytosine (5-mC) and altering transcription. Most DNA methylation occurs at the CpG site, where a cytosine nucleotide is located next to a guanidine nucleotide. When a methylated CpG island is found in the promoter region of a gene, the expression of that gene is repressed. DNA methylation is performed by a family of methyltransferase enzymes called DNA methyltransferases (DNMTs). In mammalian cells, DNMTs are divided into two major families: maintenance methyltransferase (DNMT1) and de novo methyltransferases (DNMT3A, DNMT3B, and DNMT3L). DNMT2 also displays weak methyltransferase activity [15]. A schematic representation of the DNA methylation process is presented in Figure 1.

DNA methylation is associated with imprinting, fate specification and cellular differentiation [16,17]. DNA methylation is involved to regulate gene expressions in a genomic context-dependent manner. Briefly, DNA methylation causes transcriptional repression when located within gene promoters. However, when sites are located within gene bodies and intergenic regions, it acts differently [18]. The exposure of cells to any toxicant may cause positive or negative effects. Developing and differentiating cells are more vulnerable to these effects than adult cells. A developing fetus experiences a high level of exposure because its entire body is under development during the embryonic stage. DNA methylation is amenable to environmental factors which can be passed through cell divisions and may persist throughout the entire lifetime [19]. Thus, it is assumed that ethanol exposure may cause epigenetic changes in the developing cells of the fetus. It is evidenced that DNA methylation has a potential role in the etiology of the neurobiological problems found in children with FASD and indicate a valuable epigenetic signature. The type and extent of the epigenetic changes caused by ethanol is a topic of current research. Future studies will reveal the underlying epigenetic mechanisms of ethanol exposure. In this review, we attempted to collect all available information on in utero ethanol exposure and the subsequent alterations in DNA methylation patterns. We hope that our efforts will help future researchers by offering a survey of epigenetic mechanisms under ethanol teratogenicity.

## 2. Metabolites of Ethanol and Proposed DNA Methylation Schemes

Metabolic enzymes are involved in epigenetic mechanisms. Thus, the transcriptional regulation of rate-limiting metabolic enzymes is important for controlling metabolic changes in cells. Levels of metabolites (e.g., acetyl-CoA, s-adenosyl methionine (SAM)) and metabolic hormones contribute to the regulation of gene expression. The activities of major enzymes involved in epigenetic modifications are partially regulated by the concentrations of available substrates and co-factors [20]. Thus, cellular metabolites are involved in the transcriptional regulation of genes, as well as epigenetic mechanisms. It was previously found that prenatal alcohol exposure affects one carbon metabolism and regulates functional gene expressions [21].

**Figure 2:**
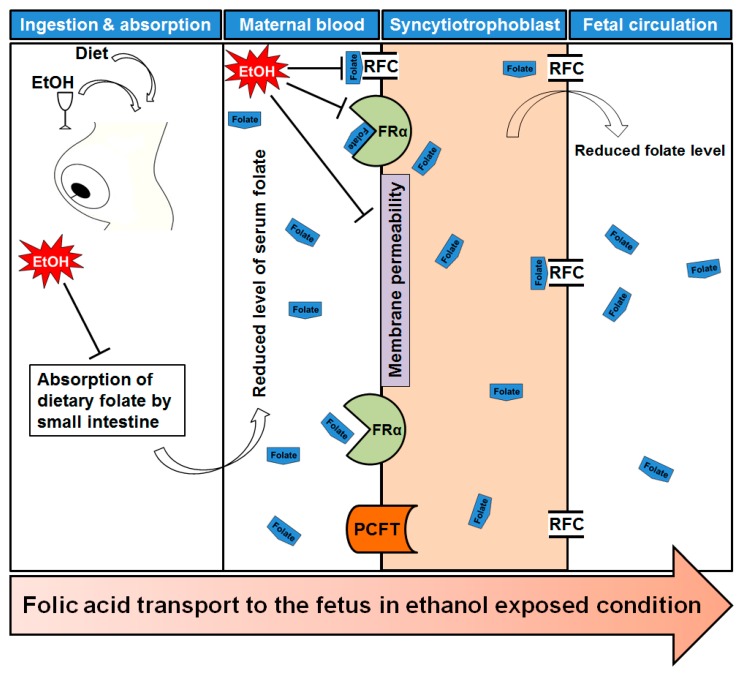
Folate transportation mechanism under ethanol exposed condition. After ingestion of alcohol it is readily absorbed through stomach and intestine. Maternal dietary folate absorption by small intestine is reduced by ethanol intake. As a result, maternal serum folate level is also affected. Transport of folates across the placental membrane is accomplished by placental folate receptors, folate receptor α (FRα) at the syncytiotrophoblast membrane. The reduced folate carrier (RFC) and the proton-coupled folate transporter (PCFT) are also involved in folate uptake in placenta. Ethanol exposure reduced the expression of FRα and RFC, and caused a significant reduction in receptor activity. Finally, there are low levels of folate in fetal circulation which may offer a reduced methylation of DNA.

Lipotropes are dietary methyl donors that play important roles in metabolism. Dietary lipotropes influence the availability of SAM and, consequently, may influence genomic DNA methylation patterns and the relative expression of nearby genes. Chronic alcoholics are known to suffer from malnutrition [22], and this lack of nutrients could affect SAM production, leading to alterations in DNA methylation patterns. In chronic alcoholics, serum folate levels are significantly reduced compared with non-alcoholics [23]. The demand of folic acid dramatically increased during pregnancy period to meet the requirement for fetal DNA synthesis and proliferation. Maternal alcohol intake reduces the serum folate level in the pregnant mother blood [24]. One of the main reasons to decrease maternal folate level is that ethanol reduces intestinal uptake of dietary folate [25]. The reduction of maternal folate level may cause poor transfer of folate molecules to fetal circulation and creates a slower production of SAM. Recently it was published that chronic alcohol exposure decreased folic acid transport to fetus in human model [26]. Chronic ethanol-exposed primary trophoblasts and BeWo cells showed reduction in mRNA expression of folate binding and transport proteins, including folate receptor α (FRα) and reduced folate carrier (RFC), respectively [25,27]. They also mentioned the possibility of affecting membrane permeability properties that permits poor transportation. A schematic diagram is presented in Figure 2 showing the folate transportation mechanism under ethanol exposed condition.

**Figure 3:**
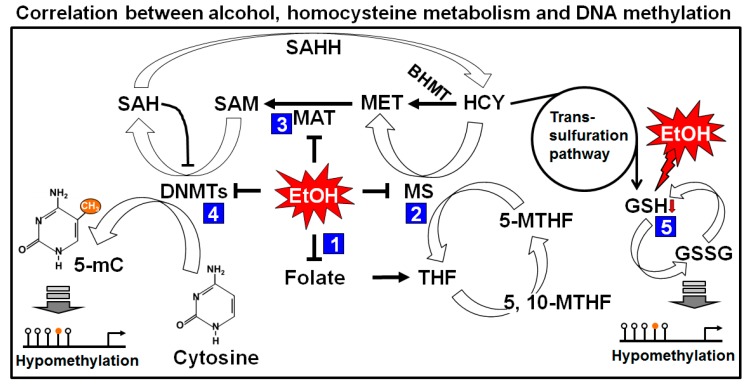
Effects of alcohol on one carbon metabolism and DNA methylation. Folic acid starts the folate cycle by converted itself to tetrahydrofolate (THF). THF is then converted to 5-methylene-THF (5-MTHF) to donate one carbon through the methylation of homocysteine (HCY) by methionine synthase (MS). The methionine cycle starts with the acceptation of the carbon from the folate cycle to form methionine (MET). s-adenosylmethionine (SAM) is formed from MET through the action of methionine adenyltransferase (MAT). SAM is further demethylated by donating the methyl group to Cytosine residue of DNA (in presence of DNMTs) and form s-adenosylhomocysteine (SAH). After deadenylation of SAH by s-adenosyl homocysteine hydrolase (SAHH), SAH is returned back to homocysteine and completes a full turn of the methionine cycle. HCY can also enter in to the transsulfuration pathway to form glutathione (GSH). Maternal heavy drinking reduces folate level (1), and cause inhibition of MS (2) and MAT (3), resulting low level of SAM. Reduction of SAM may cause induction of SAH which inhibits DNMTs (4) and causing global hypomethylation of DNA. Elevated HCY level produces GSH which is rapidly depleted by ethanol (5) and shifts the reaction from homocysteine cycle to transsulfuration pathway, causing further global hypomethylation of DNA. BHMT: betaine homocysteine methyltransferase.

Many studies have shown that folate deficiency reduces SAM levels and the SAM to s-adenosyl homocysteine (SAH) ratio and increases SAH concentrations by inactivating methionine synthase (MS) and methionine adenyltransferase (MAT) [28,29,30]. Elevated SAH concentration and ethanol itself inhibit DNMTs not to methylate DNA [31,32]. As a result it hampers DNA methylation process and causes hypomethylation. Another major candidate, glutathione (GSH) is connected to the methionine-homocystine pathway. GSH is produced from homocysteine through the transsulfuration pathway. The resulted GSH is further converted to its oxidized form, glutathione disulfide (GSSG). The ratio of GSH/GSSG is maintained by the activity of the glutathione reductase (GR) enzyme [33]. It was reported that ethanol exposure depleted the GSH level and that depletion could influence new GSH formation from homocysteine to recover the deficiency [34]. This phenomenon shifts the reactions from producing methionine and SAM to GSH production. As a result, overall reduction of DNA methylation occurred. Ethanol-mediated depletion of GSH is thought to be mediated by reactive oxygen species (ROS) because, with excessive ROS, the level of GSH was found to be depleted [35]. A schematic representation of ethanol’s effect on one carbon metabolism is summarized in Figure 3.

**Figure 4:**
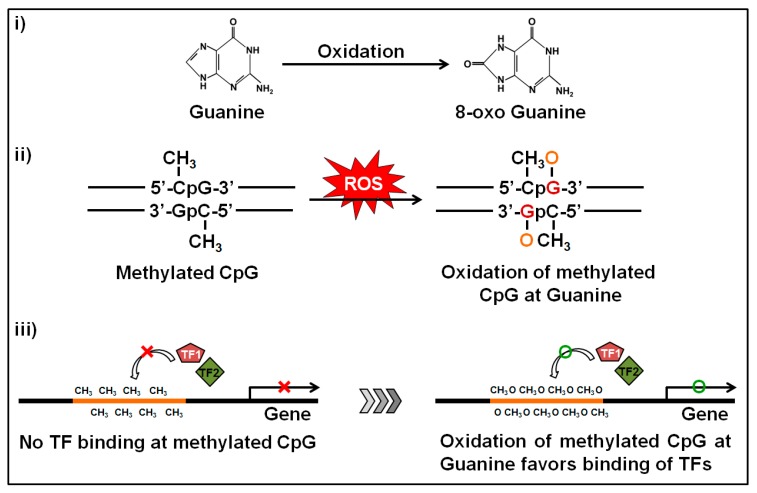
Oxidation of the methylated locus at guanine bases and subsequent impact. Guanine is the base that is more sensitive to oxidation. The oxidation produces 8-oxo deoxyguanosine from guanine and alters the structure of methylated CpGs. During oxidative stress, the methylated CpGs turns the promoter to a hydrophilic state by forming 8-oxo deoxyguanosine and facilitates the binding of transcription factors which in turn induces transcription of the related gene.

Another important phenomenon of ethanol exposure is that it produces a high amount of ROS through the oxidative pathway of ethanol metabolism. Elevated ROS levels induce intracellular oxidative stress and can alter transcriptomic and epigenetic mechanisms involved in human carcinogenesis [36,37]. Gestational oxidative stress is responsible for alteration of fetal epigenetic patterns that can lead to cardiac disorders in neonatal subjects [38]. The main targets of ROS are the CpGs and the CpG islands near by the gene promoters. Oxidative stress causes oxidation of the methylated cytosine and/or guanine bases within the CpGs. Oxidation of the methylated cytosine base produces hydroxyl methylcytosine (5-hmC) and this one is a pre-requisite for active demethylation [39]. Abnormal 5-hmC levels in cells may cause aberrant active DNA demethylation. On the other hand, oxidation of the guanine base produces 8-oxo deoxyguanosine (8-oxoG) which transforms methylated CpG islands to a more hydrophilic state to ease binding of transcription factors [40,41] (Figure 4). ROS-mediated oxidation of either methylated cytosine or guanine bases may cause hypomethylation of DNA and confer epigenetic regulation. This phenomenon is commonly found in neurodegenerative diseases [42] and seems to be the most clinically-associated consequence of high oxidative exposure [41]. Another circumstance of 8-oxoG formation is that it substantially diminishes methyl CpG binding-domain proteins (MBDs) binding when 8-oxoG is adjacent to the 5-mC and weakens the affinity bonding between DNA and DNMT3A, resulting in a reduced methylation status [43]. Furthermore, the unbound DNMT3A could result in random methylation of cytosine bases at non-CpG sites [44]. In cancer biology, ROS is associated with global hypomethylation and aberrant hypermethylation of some tumor suppressor gene promoters [45]. It was recently published that ROS can function as a catalyst that favors DNA methylation processes and caused hypermethylation of gene promoters [37]. Superoxide molecules are able to regulate DMNT functions as well [46]. When a superoxide molecule reacts with the C-5 atom it gets deprotonated sooner. On the other hand, after transferring the methyl group in SAM, it contains a positively-charged S-atom. Thus, it is possible to react with the nucleophilic C-5 atom and the charged S-atom to generate methylated cytosine. There is another possibility that the radical could react with another superoxide molecule to produce oxygen and hydroperoxyl [46]. In this way we can explain the ROS-mediated mechanism of DNA hypermethylation in promoter regions of targeted tumor suppression genes. Ethanol-mediated oxidation of guanine bases were first reported by Lia and Ohara [47] where they showed that the products of ethanol oxidation (1-hydroxyethyl and 2-hydroxyethyl radicals) were able to replace the C8 position of guanine and guanine bases in RNA and DNA [47]. A follow-up report was also published where they found the presence of C8-(1-hydroxyethyl) guanine in rat liver after ethanol exposure [48].

## 3. Evidence of the Alteration of DNA Methylation by Alcohol in Utero

Prenatal alcohol exposure was reported to cause altered expression of methyltransferase enzymes, and hypo- and hyper-methylation in gene promoters. The transient exposure of immature rodents to ethanol during postnatal day 7, comparable to a time point within the third trimester of human pregnancy, induces impaired DNA methylation by reducing the expression of two methyltransferase enzymes (DNMT1 and DNMT3A) [49]. Another report showed that administration of a low dose of ethanol to infant rats during the synaptogenesis period caused an induction of DNMT3A protein expression [50]. The craniofacial defects observed in FASD phenotypes of Japanese rice fish embryogenesis are the results of dysregulations in dnmt1 expression [51]. Prenatal binge alcohol exposure showed elevated mRNA levels of *DNMT1*, *DNMT3B*, and *MeCP2* in the pituitary gland of fetal rats [52]. Not only are the expressions of methyltransferases altered, but the activity was also reduced significantly. Acute ethanol administration to pregnant mice produced fetuses that had lower levels of DNA methylase activity even in the presence of excess SAM [53,54].

Alcohol-mediated changes in DNA methylation may affect multiple generations subsequent to alcohol exposure [54]. Children born with FASD have unique DNA methylation defects and are thought to adopt it from subsequent exposure to alcohol in utero [55,56]. Fetuses exposed to ethanol display hyper- and hypo-methylated CpGs which are related to neurodevelopmental process and diseases [19,57]. Neonatal alcohol exposure altered *VGLUT2* gene expression in the adult hippocampus by reducing the methylation signature in its promoter [58]. Ethanol was found to hamper DNA methylation in a region of the brain known as the dentate gyrus, causing developmental retardation [59]. Recently, it was published that alcohol caused alterations of methylation profiles of two maternally-imprinted loci, KvDMR1 and PEG3 DMR, in a locus-specific manner [60]. Another report showed that ethanol had the ability to modulate the developmental rhythm of DNA methylation process during embryogenesis of Japanese rice fish [61]. Alcohol-mediated hypermethylation at the *A^vy^* locus in *A^vy^* heterozygous mice [62] and hypomethylation at the *Igf2* locus in developing embryos [63] extended our knowledge about the ability of alcohol to alter developmentally-significant regions of the genome in an epigenetic manner. Bi-directional methylation changes were also reported by Liu et al. [64] who found that alcohol exposure caused neural tube defects through the alteration of DNA methylation (both hypomethylation and hypermethylation).

The similarity between epigenetic alterations mediated by alcohol and FASD etiology deserve further attention. Fetal alcohol exposure in the third trimester alters total DNA methylation in the murine hippocampus and prefrontal cortex [65]. Additionally, high throughput embryo analysis has shown that alcohol exposure alters the methylation pattern of more than one thousand genes involved in early neural development [64,66]. Zhou showed that alcohol inhibits the pathway by which moderately methylated genes become hypermethylated and hypomethylated, indicating the ability of alcohol to disrupt the methylation program of DNA [67]. A complete summary of altered methylome patterns by prenatal alcohol exposure is presented in Table 1.

In this review, we have discussed the effect of gestational alcohol exposure and subsequent alteration in fetal methylation patterns in the genomic DNA. There is another important phenomenon that exists regarding ethanol exposure which deals with the alteration of paternal sperm methylation. Though sperm methylation is not counted as a gestational event, but it is covered by the broad term “prenatal” [68]. Several reports are already published and have provided evidence that ethanol has specific effects on DMA methylation in sperm. The first report showed that intake of ethanol for nine weeks caused a reduction in DNMT1 expression in sperm [69]. In animal models, it was found that ethanol caused a significant decrease in DNA methylation at paternally-imprinted regions in sperm [70,71,72]. A similar result was also documented in humans which showed that men with moderate alcohol consumption caused reduced methylation patterns in paternally-imprinted regions in sperm [73]. To gain proper knowledge on alcohol-mediated alteration of sperm methylation, a global analysis of DNA methylation is required.

## 4. Conclusions

Maternal alcohol drinking may cause damage to the developing fetus in several aspects through direct and indirect manners. Researchers have identified various signaling pathways and molecular mechanisms which showed the mode of action of ethanol toxicity. However, the alteration of epigenetic mechanisms by ethanol is less well known. Researchers have started showing us the relevance of alcohol and DNA methylation from the last decade. The inhibition of one-carbon metabolism by ethanol is a vital path to suppression of DNA methyltransferase enzymes and the subsequent methylation status. We have gathered the known relationships between DNA methylation and ethanol-mediated teratogenesis, but additional study is needed to elucidate the correlation between these epigenetic changes and FASD.

## Figures and Tables

**Table 1:** List of altered DNA methylation status by in utero ethanol exposure.

Model Organism	Pattern of Alcohol Exposure	Sampling	Methylome Status	Reference
Mouse	3 g/kg twice a day	GD12 fetal	reduction of DNA methylation	[53]
Mouse	approximately 400 mg/dL (88 mM) in embryo culture medium	GD8.25 embryos (in vitro)	hypermethylation and hypomethylation of gene promoters	[64]
Mouse	10% *v*/*v* (in drinking water) equivalent to ~120 mg/dL in blood	P28 liver	hypermethylation at *A^vy^* locus	[62]
Mouse	5.8 g/kg (intragastrically intubated)	GD9 embryonic tissue	reduction of DNA methylation at CpG sites in the *Igf2* DMR1	[63]
Mouse	3.0 g/kg in milk (intragastrically intubated)	P21 brain	induction of methylation in the hippocampus and prefrontal cortex	[65]
Mouse	4% *v*/*v* (in liquid diet) equivalent to ∼120–160 mg/dL in blood	P7 hippocampus	reduction of both 5-mC-im and 5-hmC-im in neuroepithelium; induction of both 5-mC-im and 5-hmC-im in Conus Ammonis	[59]
Mouse	2.5 g/kg (subcutaneous injection)	P70 whole brain	at least 6660 promoter regions are differentially methylated	[56]
Mouse	10% *w*/*v* (in liquid diet) equivalent to ∼88.3 ± 11.5 mg/dL in blood	E15–17 brain followed by neural progenitor cell (NPC) culture	decreased mRNA levels of *DNMT1* and *DNMT3A* genes	[74]
Mouse	1.0 g/kg (subcutaneous injection)	P7 brain	enhancement of DNMT3A and MeCp2 protein levels	[50]
Mouse	2.5 g/kg (subcutaneous injection)	P7 brain	reduction of DNA methylation and protein level of DNMT1 and DNMT3A	[49]
Mouse	10% *v*/*v* (in drinking water) equivalent to ~120 mg/dL in blood	P28 hippocampus	CpG islands of *Olfr110*, *Vmn2r64*, *Vmn2r64*, *Vpreb2*, and *Olfr601* are highly methylated	[57]
Mouse	10% *v*/*v* in drinking water	P87 hippocampus	reduction of DNA methylation status at *Slc17a6* promoter and subsequent increase of its mRNA	[58]
Rat	6.7% *v*/*v* (in liquid diet) equivalent to 120–150 mg/dL in blood	P60-P90 pituitary gland	induction of *DNMT1*, *DNMT3b* and *MeCP2* mRNAs; CpG hypermethylation of *D2R* gene promoter	[52]
Rat	5% *wt*/*v* (in daily diet) equivalent to ~105 mg/dL in blood	GD21 brain for primary astrocyte culture	hypermethylation of the *GFAP* gene promoter both in vitro and in vivo	[75]
Rat	6.0 g/kg per day	GD21 and P10 olfactory bulbs	hypermethylation of *BDNF* gene	[76]
Rat	6.7% *v*/*v* (in liquid diet) equivalent to ~120–150 mg/dL in blood	P60–65 brain	induction of DNMT1 and MeCp2 protein expression; hypermethylation of *POMC* gene and reduced mRNA expression of *POMC*	[77]
Rat	4.5 g/kg in distilled water throughout whole gestetion followed by 3.0 g/kg of ethanol in enriched milk for newborn pups	PD 21 hippocampus	enhancement of DNMT enzyme activity	[78]
Japanese rice fish	300 mM in vitro	embryogenesis (2–6 day-post-fertilization, dpf)	reduction of *DNMT1* mRNA at 2 dpf but causes induction at 6 dpf	[51]
Japanese rice fish	300 mM in vitro	embryogenesis (6 day-post-fertilization, dpf)	elevated expression of MBP mRNAs (*MBD1B*, *MBD3A*, *MBD3B*, *MECP2*)	[61]
Young children	clinically diagnosed with FASD	3–6 years old males, buccal epithelial cells	CpGs are differentially methylated	[55]
Young children	clinically diagnosed with FAS	1–16 years, blood and buccal epithelial cells	reduction of DNA methylation at the PEG3 DMR and KvDMR1 loci	[60]
Young children	clinically diagnosed with FASD	5–18 year olds, buccal epithelial cells	658 differentially methylated sites are identified	[19]
Neural stem cell (NSC) culture	86.8 mM (400 mg/dL) in culture medium	48 h of exposure	induction of methylation status of genes related to cell cycle progression	[79]
NSC culture	400 mg/dL (88 mM) in vitro	differentiating neurospheres	reduction of methylation status in NSC genes.	[66]
Mouse embryonic fibroblasts	25 or 200 mM	cells are exposed for 24 h	impaired DNA methylation status and reduced DNMT1, DNMT3A and DNMT3B proteins expression	[80]
EB	20 or 50 mM	embryoid bodies (EB) exposed for 24 or 48 h	global DNA methylation changes at the transcription start site (TSS) and CpGs	[81]

GD, gestational day; P, postnatal day.
